# Strontium isotope stratigraphy through the Flatreef PGE-Ni-Cu mineralization at Turfspruit, northern limb of the Bushveld Igneous Complex: evidence of correlation with the Merensky Unit of the eastern and western limbs

**DOI:** 10.1007/s00126-020-01006-3

**Published:** 2020-08-20

**Authors:** Cédric C. Mayer, Pedro J. Jugo, Matthew I. Leybourne, Danie F. Grobler, Alexandre Voinot

**Affiliations:** 1 Mineral Exploration Research Centre, Harquail School of Earth Sciences, Laurentian University, Sudbury, Canada; 2 Queen’s Facility for Isotope Research (QFIR), Department of Geological Sciences and Geological Engineering, Queen’s University, Kingston, Canada; 3 McDonald Institute, Canadian Particle Astrophysics Research Centre, Department of Physics, Engineering Physics & Astronomy, Queen’s University, Kingston, Ontario Canada; 4 Ivanplats (Pty) Ltd, Mokopane, South Africa

**Keywords:** Bushveld Igneous Complex, Merensky Reef, Flatreef, Platreef, Strontium isotope stratigraphy, PGE

## Abstract

The origin of PGE-Ni-Cu mineralization in the Platreef, northern limb of the Bushveld Igneous Complex (BIC), and the possible correlation with the Merensky Reef in the eastern and western limbs has been long debated. The Platreef and Merensky Reef share the same stratigraphic position in the uppermost part of the Upper Critical Zone (UCZ), near the transition to the overlaying Main Zone (MZ). However, discrepancies in interpretations have been difficult to resolve due to the effects of intense magma-country rock interaction throughout most of the northern limb succession. To address this problem, we generated a detailed stratigraphic profile of the initial strontium isotopic ratio [Sr_i_ = (^87^Sr/^86^Sr)_i_] in plagioclase across a Flatreef interval lacking macroscopic evidence of country rock assimilation. The in situ Sr isotopic ratios in plagioclase were determined using LA-MC-ICP-MS analysis on 37 samples from a drill core (UMT094) at the Turfspruit project. Strontium isotope stratigraphy is useful because of a well-documented shift in Sr_i_ near the base of the Merensky Unit in the eastern and western limbs. The results show the existence of a significant shift (from Sr_i_ = 0.7060 to Sr_i_ = 0.7090) that matches the isotopic shift documented through the Merensky Unit in the eastern and western limbs. Thus, this new Sr isotope data indicates that the main mineralized interval of the Flatreef can be stratigraphically correlated to the Merensky Reef in the remainder of the BIC. In addition, we interpret these results as compelling evidence to suggest that the main mineralization processes in the Flatreef were likely similar to those operating in the eastern and western limbs and that interaction with local country rocks was not a necessary condition.

**Electronic supplementary material:**

The online version of this article (10.1007/s00126-020-01006-3) contains supplementary material, which is available to authorized users.

## Introduction

The Bushveld Magmatic Province (BMP) in northern South Africa encompasses an extrusive sequence (the Rooiberg Group) and an intrusive sequence (the Bushveld Igneous Complex (BIC); e.g., Kinnaird et al. 2004). The BIC is the largest layered mafic intrusion in the world (ca. 66,000 km^2^) and also hosts the largest reserves of platinum-group elements (PGE; Maier et al. 2013; Zientek et al. 2014; Zeh et al. 2015; Zientek et al. 2017). The BIC intruded into the Rooiberg Group, Transvaal Supergroup, and Archean basement, and it is composed of three main suites: (1) the Rustenburg Layered Suite (RLS), a sequence of mafic to ultramafic layered rocks; (2) the Lebowa Granite Suite, a series of granites overlying the RLS; and (3) the Rashoop Granophyre Suite, which has been interpreted as the late felsic phase of the BIC (Kinnaird et al. 2004; Kruger 2005). There are several limbs to the BIC of which the most relevant are the eastern, western, and northern limbs. The generalized magmatic stratigraphy of the RLS has been defined from studies predominantly in the eastern and western limbs and has five main zones: Marginal Zone (MaZ), Lower Zone (LZ), Critical Zone (CZ), Main Zone (MZ), and Upper Zone (UZ). Three stratigraphic intervals contain most of the PGE mineralization: the Merensky Reef and the UG-2 chromitite in the eastern and western limbs, and the Platreef in the northern limb (Mungall and Naldrett 2008; Cawthorn 2010; Peck and Huminicki 2016). In the eastern and western limbs, the Merensky Reef is located near the top of the Upper Critical Zone and almost immediately below the base of the Main Zone. Although PGE-Ni-Cu mineralization in the Platreef is located roughly at the same stratigraphic position, consensus about possible correlation between the Merensky Reef and the mineralized intervals in the Platreef has been difficult to reach, mostly because of the complexities caused by intense magma-country rock interactions in outcrops and relatively shallow drilling intersections. Recent studies (Yudovskaya et al. 2017a, b; Grobler et al. 2019) present compelling evidence for correlation between the upper parts of the Upper Critical Zone (from the UG-2 to the contact to the Main Zone) in all the three main limbs based on deep intersections of Platreef (referred to as Flatreef). Previous studies in the western and eastern limbs (Kruger and Marsh 1982; Kruger 1994; Seabrook et al. 2005) showed that stratigraphic variations in Sr isotopic ratio form a consistent marker near the base of the Merensky Unit. Recent studies (Yudovskaya et al. 2018; Beukes et al. 2020) show that similar features may be present in the northern limb. Here, we test the potential correlation of the Merensky Reef in the eastern and western limbs with the upper part of the Flatreef by documenting a detailed Sr isotopic stratigraphy through a drill core intersection of the Flatreef. The Sr isotopic data is complemented with data on the An content in plagioclase and whole rock major and trace element geochemistry.

## Geological background

### Generalized magmatic stratigraphy of the RLS with emphasis on PGE mineralization

The generalized geology of the RLS of the BIC is based on reference sections mostly within the eastern and western limbs (Von Gruenewaldt et al. 1985; Kruger 2005; Barnes et al. 2010; Maier et al. 2013; Zientek et al. 2014). Only a brief summary is presented here. The Marginal Zone (MaZ) is a unit of variable thickness (from 0 to roughly 800 m) consisting of fine-grained gabbronorites and pyroxenites, considered to be the quenched products of the intruded magmas against the floor rocks. It is divided into three suites: B1, B2, and B3, depending on whether it is in contact with the LZ, CZ, or MZ, respectively. This sequence also contains xenoliths of dolostone, quartzite and anorthosite. The Lower Zone (LZ) is mainly composed of harzburgite, dunite and orthopyroxenite with minor norite layers. It is up to 1300 m thick, but the thickness is variable due to variations in floor topography (Wilson 2012). The LZ contains less than 1% chromite through the magmatic stratigraphy and the appearance of chromitite seams is considered as the main marker of the Critical Zone (CZ). The chromite layers are grouped into three distinct clusters: up to seven Lower Group (LG) chromitites, up to four Middle Group (MG) chromitites, and up to three Upper Group (UG) chromitites. The CZ is divided into the Lower Critical Zone (LCZ) and the Upper Critical Zone (UCZ). The LCZ is dominantly pyroxenitic with some olivine-rich intervals (Kruger 2005). The appearance of an anorthosite layer between the MG2 and MG3 chromitites is commonly used as the marker to separate the LCZ from the UCZ. The UCZ is dominantly noritic and pyroxenitic and contains the PGE-rich UG-2 chromitite and Merensky Reef. The UG-2 chromitite is a massive chromitite seam (0.5 to 1.2 m thick), hosting significant PGE mineralization (e.g., ca. 2000 ppb Pt, 1200 ppb Pd, 360 ppb Ru, and 100 ppb Ir over 66 cm; Maier and Barnes 2008). The Merensky Reef (which is located in the uppermost UCZ, few 100 m above the UG-2) is not a well-defined lithological unit but a mining operation term for the roughly one meter thick zone containing the best PGE grades (Cawthorn et al. 2002). However, the Merensky Reef is typically characterized by the presence of one or two thin (few mm thick) chromitite seams, commonly with an anorthosite below the lower chromitite seam, pegmatoidal pyroxenite (or norite) between the two chromitites and non-pegmatoidal pyroxenites above the upper chromitite seam (Barnes and Maier 2002; Godel et al. 2007). Despite the variations in lithologies, the thin chromitite seams, in particular, and the high PGE grades can be traced around much of the eastern and western limbs of the BIC. Two weakly mineralized units are located near the Merensky Reef: the Pseudoreef below the Merensky Reef and the Bastard Reef above it (Maier et al. 2013).

A distinct mottled anorthosite several meters to 10s of meters above the Merensky Reef is typically used as the marker for the transition between the CZ and the Main Zone (MZ). The two to three km thick MZ consists dominantly of gabbronorite-norite sequences. Above the MZ, the first appearance of magnetitite layers is used to mark the boundary between the MZ and the Upper Zone (UZ), which is typically between 1 and 2 km thick and dominated by layers of gabbronorite, anorthosite, diorite, and magnetitite.

The general stratigraphy of the RLS in the northern limb is described in detail in the introduction to this thematic issue (Maier et al. 2020) and only the most relevant aspects are summarized here. There are a few key features to emphasize. First, the UZ and MZ are broadly similar in thickness and lithologies as in the eastern and western limbs of the BIC. Second, the CZ of the northern limb is in direct contact with floor rocks and shows much reduced thickness. Third, the interaction of Bushveld magmas with the floor rocks (metasedimentary rocks on the south, gneisses towards the north) resulted in a complex sequence of intrusive mafic-ultramafic rocks with metasedimentary xenoliths and, in most places, lack of recognizable magmatic stratigraphy, which is known as the Platreef (van der Merwe 1976; Gain and Mostert 1982; Kinnaird and McDonald 2005). Fourth, the Platreef contains PGE mineralized intervals, which can be much thicker than the PGE reefs in the eastern and western limbs (Grobler et al. 2019). A problem with the term “Platreef” is that it has been used to describe both the complete sequence of magma-country rock interaction between the Main Zone and the country rocks (up to 400 m thick) as well as the localized PGE mineralization intervals within that package. To avoid this problem, we use “Platreef Unit” for the complete sequence and “Platreef” for the PGE-Ni-Cu mineralized intervals within that unit as suggested by Mitchell and Scoon (2012) and Maier et al. (2020). In the Turfspruit area, the Platreef Unit sequences, which typically dip to the SW, have a change in slope and become subhorizontal at ca. 700 m depth (Maier et al. 2020). Such interval, known as the Flatreef, is also characterized by a decreasing amount of contamination and better preservation of magmatic stratigraphy (Grobler et al. 2019).

The lack of continuity in magmatic stratigraphy caused by complex interaction with country rocks has been one of the main limitations in establishing a correlation between the northern limb and the eastern and western limbs. Because of this, several correlation schemes have been used and there is no consistent terminology. For example, the A, B, C reef nomenclature (Barton et al. 1986; Kruger 2010); the Grasvally norite-pyroxenite-anorthosite (GNPA) member, used south of the Ysterberg-Planknek Fault (McDonald et al. 2005); the units 1, 2, and 3 used at the Aurora Project (McDonald et al. 2017). Most of these units correspond stratigraphically with the UCZ-MZ transition in the eastern and western limbs of the BIC. The exception seems to be ultramafic to mafic units north of the Hout River shear zone, which have been interpreted to represent a structurally separated compartment of RLS magmas (Kinnaird et al. 2017).

### Stratigraphy of the Flatreef at the Turfspruit project area

**Fig. 1:**
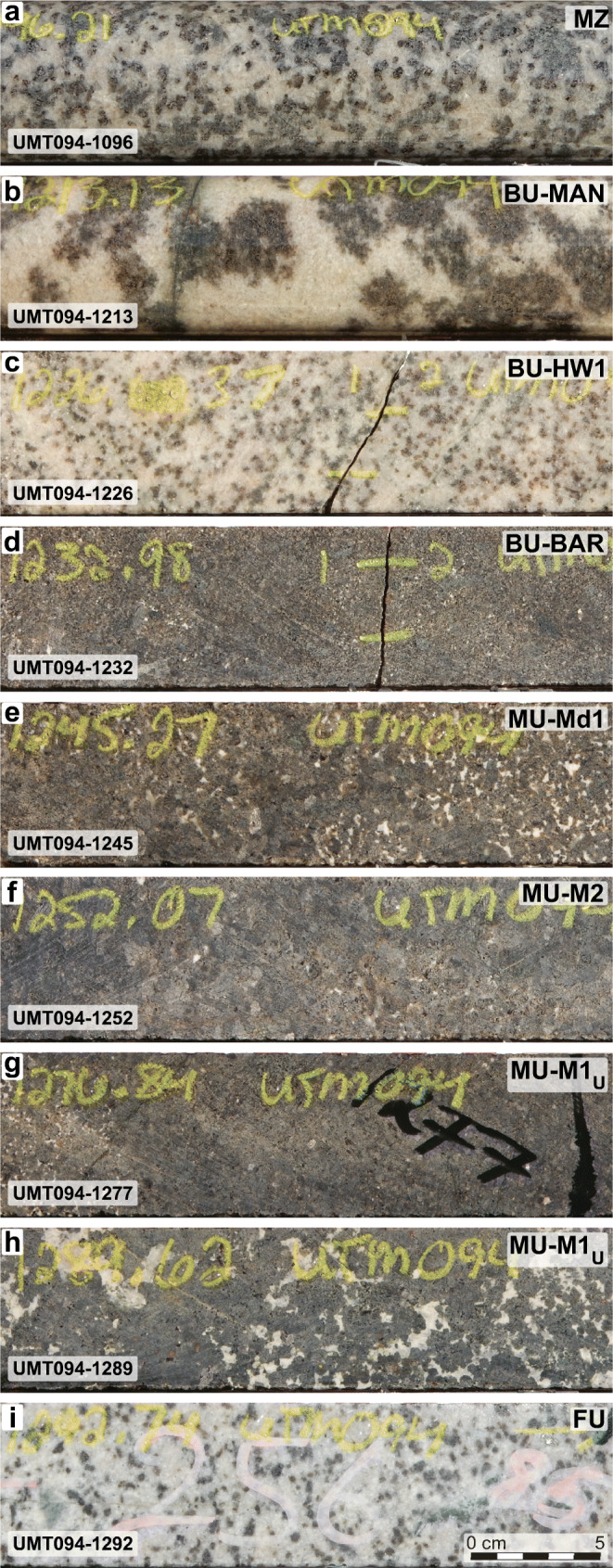
Examples of the main rock types analyzed in this study. The codes for the main units and sub-units are indicated in the upper right corner. The sample number (drillcore ID - depth) is indicated in the lower left corner. All the pictures are from longitudinal sections of NQ core (47.6 mm diameter). A scale bar is shown on the last image. **a** Gabbronorite of the Main Zone. **b** Mottled anorthosite (MAN), the topmost sub-unit of the Bastard Unit. **c** Norite (HW1 of the BAR). **d** Bastard pyroxenite (BAR). **e** Unmineralized feldspathic pyroxenite (Md1). **f** Mineralized pyroxenite (M2). **g** Medium-grained pyroxenite that alternates with pegmatoidal pyroxenite in the M1_U_ sub-unit. **h** Pegmatoidal pyroxenite that defines the M1_U_ sub-unit. **i** Norite at the top of the FU and immediately below the MU. MZ = Main Zone, BU = Bastard Unit, MU = Merensky Unit, FU = Footwall Unit

The stratigraphy of the Flatreef was described in Yudovskaya et al. (2017a, b) and Grobler et al. (2019), and only key aspects are reiterated here. Yudovskaya et al. (2017b) recognized sections of the Flatreef that were largely undisturbed and, although much thicker, shared characteristics common to the Merensky and Bastard reefs in the eastern and western limbs. They labeled the reefs as “Main” and “Upper” and concluded that they correlate with the Merensky and Bastard reefs, respectively, but argued that the correlation does not imply lateral connectivity. Grobler et al. (2019) recognized four major units below the MZ that are correlative of UCZ units in the eastern and western BIC: Bastard Cyclic Unit (BCU), Merensky Cyclic Unit (MCU), Footwall Cyclic Unit (FCU), and UG-2 Cyclic Unit (UG2CU). We are adopting this nomenclature but without the “cyclic” qualifier for simplicity and to avoid the genetic connotations of the term as discussed in Irvine (1982) and Hunt et al. (2018). Figure 1 shows representative examples of some of the main lithologies, obtained from the drill core used in this study (UMT094). At the top of the studied sequence is the Main Zone (MZ) consisting of medium grained gabbronorite (Fig. 1a). The contact to the Bastard Unit (BU) of the CZ is defined by a thick interval of mottled anorthosite (MAN sub-unit; Fig. 1b). The MAN is underlain by an interval of interlayered norite and pyroxenite (Fig. 1c), which constitutes the hanging wall (HW1) of a weakly mineralized feldspathic pyroxenite (Fig. 1d), correlative of the Bastard Reef (BAR) in the western and eastern limbs of the BIC. In some drill cores, there is a very thin chromitite at the base of the BAR.

Below the BU is the Merensky Unit (MU). Its upper portion is labeled the Middling Sub-unit (Md; Fig. 1e), which consists of interlayered pyroxenite-norite sequences that vary in thickness, can be absent and have very low PGE-Ni-Cu content. The main mineralized interval (cf. the “Main Reef” in Yudovskaya et al. 2017b) is divided into sub-units based on textural and mineralogical differences identifiable in drill core. The top portion (“Merensky 2”or M2) consists of a medium-grained feldspathic orthopyroxenite (Fig. 1f). The bottom portion (“Merensky 1” or M1) is dominantly pegmatoidal and consists of two different lithologies: an orthopyroxenite (“M1 Upper” or M1_U_) and a feldspathic harzburgite (“M1 Lower” or M1_L_). Although the “upper” and “lower” descriptors reflect the typical spatial relationship, the presence of olivine is the defining feature for the nomenclature and the M1_L_ occurs stratigraphically above the M1_U_ in some places (Maier et al. 2020). The M1_U_ orthopyroxenite contains non-pegmatoidal patches (Fig. 1g) that grade into more abundant pegmatoidal textures (Fig. 1h) and the transition between M2 and M1_U_ is not always sharply defined.

The Footwall Unit (FU) to the MU is composed of interlayered pyroxenite and norite (Fig. 1i). In most of the Flatreef area the FU was affected by various degrees of interaction of BIC magmas with the country rocks, which created intervals with complex textures and lithologies that are labeled as the Footwall Assimilation Zone (FAZ). The extent of local crust assimilation is less pronounced towards the deepest part of the project, where the correlatives of the UG-2 chromitite seam can be identified (the “UG-2 equivalent” or “UG-2E” in Langa et al. 2020). In those cases, the UG-2 Unit (UG2U) is defined as UG-2 chromitite and its hanging wall and footwall (UG2HW, UG2FW).

### Strontium isotopic stratigraphy through the RLS

Strontium isotope stratigraphy has been widely used in layered mafic intrusions to distinguish isotopically different magmatic sequences, which could be used for stratigraphic correlations across the intrusion, but also for interpretations of magmatic processes. The Sr isotope framework of reference for the BIC has been established from sections of the eastern and western limbs (Hamilton 1977; Kruger and Marsh 1982; Seabrook et al. 2005; Karykowski et al. 2017). The northern limb, in contrast, has received less attention. A summary of studies documenting the Sr isotope stratigraphy throughout the RLS (from Hamilton 1977 to Beukes et al. 2020) is provided in the supplementary materials (ESM1 Table 1). The most relevant observation is the existence of a major shift in Sr_i_ values at the top of the UCZ that roughly coincides with the Merensky Unit [Sr_i_ = (^87^Sr/^86^Sr)_i_]. Kruger and Marsh (1982) showed that, in the Rustenburg area, Sr_i_ = 0.70636 ± 0.00003 (*n* = 5) through the FU immediately below the MU but increase to up to Sr_i_ = 0.70740 through the 10 m thickness of the MU and then stabilize to Sr_i_ = 0.70765 ± 0.00020 (*n* = 6) through the BU. Seabrook et al. (2005) compiled similar trends for other locations in the eastern and western limbs and showed that the Sr_i_ shift correlates with a change in Sr content in plagioclase, with plagioclase from the Critical Zone having > 450 ppm Sr (and Sr_i_ < 0.7065) and plagioclase from the Main Zone having < 400 ppm Sr (and Sr_i_ > 0.7075). They suggested that the stratigraphic interval containing the MU and BU, over which the Sr_i_ shifts occurs, represents a zone of interaction of magmas from the UCZ and MZ and should be considered a Transitional Unit. Yang et al. (2013) documented (^87^Sr/^86^Sr)_i_ data from in situ analyses in plagioclase in 11 selected samples from the Upper Critical Zone of the Union Section (western limb). Although their results are broadly consistent with previous results using whole rock, they also found significant variations in An content, from An_55_ to An_72_ in Merensky samples, and in Sr isotopic ratio within and between grains of in several samples (e.g., 0.70506 < Sr_i_ < 0.70666 in UG1 samples). They attributed such variations to two processes: accumulation of plagioclase crystals from magmas of different Sr isotopic ratio, followed by late-stage percolation of residual melts from a different stratigraphic level. Karykowski et al. (2017) expanded on the work of Yang et al. (2013) and used in situ Sr isotope compositions of plagioclase to create a composite reference profile of Sr_i_ variations through the entire RLS stratigraphy using samples from the Union Section of the western limb (LZ to the MZ) and UZ samples from the northern limb. This reference profile highlights again that the most significant shift in Sr_i_ is the one documented by Kruger and Marsh (1982) through the MU.

In contrast to the eastern and western limbs, fewer studies on Sr isotopic stratigraphy have been completed in the northern limb. Early work by Cawthorn et al. (1985) and Barton et al. (1986) recorded large variations in the Platreef (0.7054 < Sr_i_ < 0.7227) at Overysel and Sandsloot. Kruger (2005) measured Sr isotopes in plagioclase and orthopyroxene separates from relatively shallow drillcore (up to 253 m) on the Turfspruit farm. Their plagioclase data yield an average Sr_i_ = 0.71103 ± 0.00168, which is significantly higher than the reference isotopic values from the eastern and western limbs. Because the Sr_i_ values are shifted towards the range of gneisses, tonalitic veins, and dolostone (Sr_i_ > 0.720), they have been interpreted to record contamination with local country rocks (Cawthorn et al. 1985). More recently, Mangwegape et al. (2016) documented the variations in Sr_i_ through the Main and Upper zones in the northern limb. Because of significant variations in Sr_i_ in the lower part of the Main Zone, they concluded that the lower MZ was constructed by repeated influxes of magmas. Huthmann et al. (2017) showed that there are no significant variations (0.7065 < Sr_i_ < 0.7075) throughout the magmatic stratigraphy at the Waterberg Project and interpreted the differences to the data in Mangwegape et al. (2016) as evidence of a different magmatic basin across the Hout River Shear Zone. Yudovskaya et al. (2018) document Sr_i_ in plagioclase (0.7075 < Sr_i_ < 0.7087) from 3 samples of the upper part of the Flatreef (Main and Upper reefs) that is comparable to the range in Sr_i_ for the MU and BU at the Union Section (Yang et al. 2013) and concluded that the mineralized interval correlates with the Merensky Reef. Beukes et al. (2020) investigated the Sr isotopic stratigraphy at the Macalacaskop farm. Their results also show an increase in Sr_i_ (from Sr_i_ = 0.707 to Sr_i_ > 0.709) that crudely matches the isotopic shift described for the eastern and western limbs, supporting the correlation of the upper parts of the Platreef with the UCZ-MZ transition in the eastern and western limbs of the BIC. Thus, the main limitation of early studies in the northern limb was the lack of suitable samples due to the complex interaction of the Bushveld magmas with country rocks and the main limitation of the most recent work is the poor resolution of the Sr_i_ variations.

## Materials and methods

Some of the deepest drill cores available from the Turfspruit area show well-preserved magmatic stratigraphy through the intervals with PGE mineralization, with no macroscopic evidence of contamination from local country rocks. The samples analyzed were collected from drill core UMT094. This hole was selected because core logging showed well-preserved magmatic stratigraphy through PGE-Ni-Cu mineralization. In addition, detailed S isotope data from UMT094 (Keir-Sage et al. 2020) show that δ^34^S values across the mineralized intervals (δ^34^S < 4 ‰) are indistinguishable from those documented in the eastern and western limbs of the BIC (Magalhães et al. 2018) making those samples ideal for detailed Sr isotopic stratigraphy in the northern limb.

Hole UMT094 is 1602 m long and intersects 1185 m of MZ gabbronorite before intersecting mineralized units (BU and MU). Thirty-six samples were selected based on lithology, the lack of any recognizable alteration or assimilation textures in hand specimen, and distance to the interpreted base of the MU, as identified by significant increase in PGE content in whole-rock assays and the existence of a thin chromitite stringer. Because the main focus of the project was to document the possible existence of shifts in ^87^Sr/^86^Sr_i_ across the MU, sample density was higher than in previous studies (on average one sample every 4.5 m) including the top 25 m of the FU below the MU, the entire MU, and the first 15 m of the BU above the MU (including the BAR). Standard polished sections (30 μm) were prepared at Laurentian University for petrographic analysis. To ensure enough material could be ablated during in situ Sr isotope analyses, a matching set of thicker polished sections (100 μm) were prepared from the same billets.

Samples were sent to ALS Geochemistry, Vancouver, BC, Canada, for whole-rock geochemical analyses including (1) major elements by ICP-AES following lithium metaborate fusion; (2) trace elements by ICP-MS following lithium metaborate fusion to include elements within phases resistant to acid digestion (zircon, chromite, monazite); (3) trace and some major elements by an ultra-trace four-acid digestion (HF, HClO_4_, HCl, HNO_3_) followed by a mixture of ICP-AES and ICP-MS analysis, to allow lower detection limits on elements not incorporated in resistant phases; and (4) Au, Pt, and Pd contents by lead oxide fire assay with subsequent analysis by ICP-MS and ICP-AES. Reference materials (MRG-1 and SY-3) were sent for quality control.

Petrographic observations were completed to identify and document mineralogy, alteration, veining, grain size, and shape, and to select areas of interest for later in situ plagioclase analyses. Plagioclase composition was determined by electron probe microanalysis (EPMA) using a Cameca SX-100 at the Geoscience Laboratories (GeoLabs) of the Ontario Geological Survey, Sudbury, Ontario, Canada. Plagioclase grains selected from 24 samples, representative of the magmatic stratigraphy, were analyzed. At least seven analyses were collected on different grains per sample, avoiding grains or zones with evident alteration. Analytical conditions used for wavelength-dispersive X-ray spectroscopy (WDS) comprised a beam diameter of 8 μm, probe current of 20 nA, and acceleration voltage of 20 kV. Elements analyzed (reported as oxides) and detection limits (in wt.%) were SiO_2_ (0.024), TiO_2_ (0.018), Al_2_O_3_ (0.019), MgO (0.009), CaO (0.018), MnO (0.027), FeO (0.026), SrO (0.071), Na_2_O (0.012), and K_2_O (0.014).

Element distribution maps were acquired with laser ablation inductively coupled plasma mass spectrometry (LA-ICP-MS) on selected plagioclase grains to assess the effects of alteration on trace element homogeneity (especially for Rb and Sr). Samples and areas of interest were selected based on petrographic observations of plagioclase type (cumulus or interstitial) and grains that contained fresh and altered sectors. Data were collected at Laurentian University using a Resonetics-M50 excimer laser (193 nm) coupled with Thermo X-SeriesII quadrupole ICP-MS using parallel lines in rastering mode (Ulrich et al. 2009) and the following operational parameters: laser energy of 4 J/cm^2^, pulse frequency of 8 Hz, laser beam diameter of 36 μm, and scan velocity of 18 μm/s. Glass standards (GSC-1, GSE-1G, GSD-1G, and NIST610) were used for calibration and quality control. The masses analyzed correspond to ^23^Na, ^24^Mg, ^27^Al, ^29^Si, ^39^K, ^44^Ca, ^45^Sc, ^47^Ti, ^51^V, ^52^Cr, ^55^Mn, ^57^Fe, ^60^Ni, ^71^Ga, ^72^Ge, ^85^Rb, ^88^Sr, ^89^Y, ^90^Zr, ^133^Cs, ^137^Ba, ^139^La, ^140^Ce, ^141^Pr, ^146^Nd, ^147^Sm, ^153^Eu, ^157^Gd, ^159^Tb, ^163^Dy, ^165^Ho, ^166^Er, ^169^Tm, ^172^Yb, ^175^Lu, ^204^Pb, ^206^Pb, ^207^Pb, and ^208^Pb.

In situ analyses of Sr isotopes in plagioclase were based on the procedures documented in Yang et al. (2013), Mangwegape et al. (2016), Karykowski et al. (2017), and Wilson et al. (2017). Plagioclase grains (rim, core, or whole plagioclase in relatively small grains) were selected on polished sections 100 μm thick. In situ Sr isotope analyses were performed by laser ablation multi-collector inductively coupled plasma mass spectrometry (LA-MC-ICP-MS) at the Queen’s Facility for Isotope Research (QFIR) using a 193 nm excimer laser (Elemental Scientific NWR193) interfaced with a Thermo-Finnigan Neptune MC-ICP-MS. A laser beam of 150 μm diameter was used with a repetition rate of 10 Hz, a beam energy density of 2.3 J/cm^2^, and a duration of 120 s per analysis preceded by a 60 s blank analysis. The masses analyzed correspond to ^82^Kr, ^83^Kr, ^84^Sr, ^85^Rb, ^86^Sr, ^87^Sr, ^88^Sr, ^44^CaPO, as well as doubly charged REE (^163^Dy^++^, ^167^Er^++^, ^171^Yb^++^, ^173^Yb^++^, and ^175^Lu^++^) using dynamic mode (centre mass jumping from 86 to 86.5). The idle time was set to 3.0 s to allow for magnet and amplifiers to settle. The integration time was set to 2.0 s for Kr, Rb, Sr, and CaPO, and 1.0 s for doubly charged REE. After analysis, all data that resulted in negative values were nulled (mainly Kr and REE). Five to ten spot analyses were completed per sample with one reference material (BHVO-2G, BIR-1G or TB-1G) analyzed after every two plagioclase analyses. To assess possible zonation, analyses were conducted on the rim or core domains of plagioclase grains, commonly both on the same grain if the grains were large enough. If the grains were too small to analyze the rim and core, the points were labeled as “whole plagioclase”. After acquisition, data were corrected for Kr interference (^84^Kr on ^84^Sr and ^86^Kr on ^86^Sr, calculated from ^82^Kr and ^83^Kr) using the blank analysis for each individual sample (background counts), and then corrected for doubly charged REE interference on Rb and Sr (^85^Rb was corrected for interference of ^170^Er^++^ and ^170^Yb^++^; ^86^Sr was corrected for interference of ^172^Yb^++^; ^87^Sr was corrected for interference of ^174^Yb^++^; and ^88^Sr was corrected for interference of ^176^Yb^++^ and ^176^Lu^++^). After the blank subtraction, REE^++^ correction and Kr correction on ^84^Sr and ^86^Sr, a mass bias fractionation factor was calculated using the measured ^86^Sr/^88^Sr, an exponential law and the natural ^86^Sr/^88^Sr value of 0.1194 (Russell’s law; Russell et al. 1978). The ^87^Sr/^86^Sr values were then corrected for interference of ^87^Rb on ^87^Sr. This correction was completed using the ^85^Rb/^88^Sr and ^87^Sr/^86^Sr measured, as well as the ^87^Sr/^86^Sr certified value for two of the SRM (TB-1G and BHVO-2G) analyzed at the start and end of each analytical session (typically four samples). The SRM BIR-1G was not used for the correction because of the lower Sr-Rb concentrations (which yield larger analytical uncertainties), but it was used to assess the accuracy of the procedure. The ^87^Rb/^86^Sr certified values for TB-1G were used to correct for ^87^Rb/^86^Sr in the samples using natural isotopic ratios and average concentrations of Sr (1322 ± 52 ppm) and Rb (140 ± 10 ppm) in TB-1G from peer-reviewed literature (Norman et al. 2004; Elburg et al. 2005; Lucassen et al. 2011; Kimura and Chang 2012; Norman et al. 2016). Based on the difference between the measured value and the published ^87^Rb/^86^Sr values in TB-1G, empirical correction factors were calculated for each analysis (~ 2.3 to 2.0) and applied to the ^87^Rb/^86^Sr measured. The initially calculated mass bias correction for ^87^Sr/^86^Sr was inaccurate because of ^87^Rb isobaric interference on ^87^Sr. However, there is a linear relationship between the mass bias corrected ^87^Sr/^86^Sr and the ^85^Rb/^88^Sr ratio. Thus, the sample with the highest ^85^Rb/^88^Sr ratio (TB-1G) was used routinely to determine the parameters of the linear correlation for every batch of samples and other SRM. In total, 242 analyses were completed on standard reference materials BHVO-2G, BIR-1G, and TB-1G (131, 55, 56, respectively). Estimated ^87^Sr/^86^Sr (mean and 1σ STD) are consistent with preferred values (Jochum et al. 2007; GeoReM database: http://georem.mpch-mainz.gwdg.de). The ^87^Sr/^86^Sr for BHVO-2G was 0.70347 ± 0.00022 (preferred value = 0.703469 ± 0.000007), for BIR-1G was 0.7029 ± 0.0017 (preferred value = 0.703105 ± 0.000006), and for TB-1G was 0.70565 ± 0.00011 (reference value = 0.70558 ± 0.000023, 2σ by TIMS; 0.70576 ± 0.0003 by LA-ICP-MS). The uncertainties on BIR-1G are slightly higher because of the lower Sr and Rb contents. Uncertainties were estimated during each analytical session based on the reproducibility of the standards within that sequence. The (^87^Sr/^86^Sr)_i_ was calculated using an age of 2054.89 ± 0.37 Ma (Zeh et al. 2015) and a decay constant of 1.39 × 10^−11^ (Nebel et al. 2011).

## Results

**Fig. 2:**
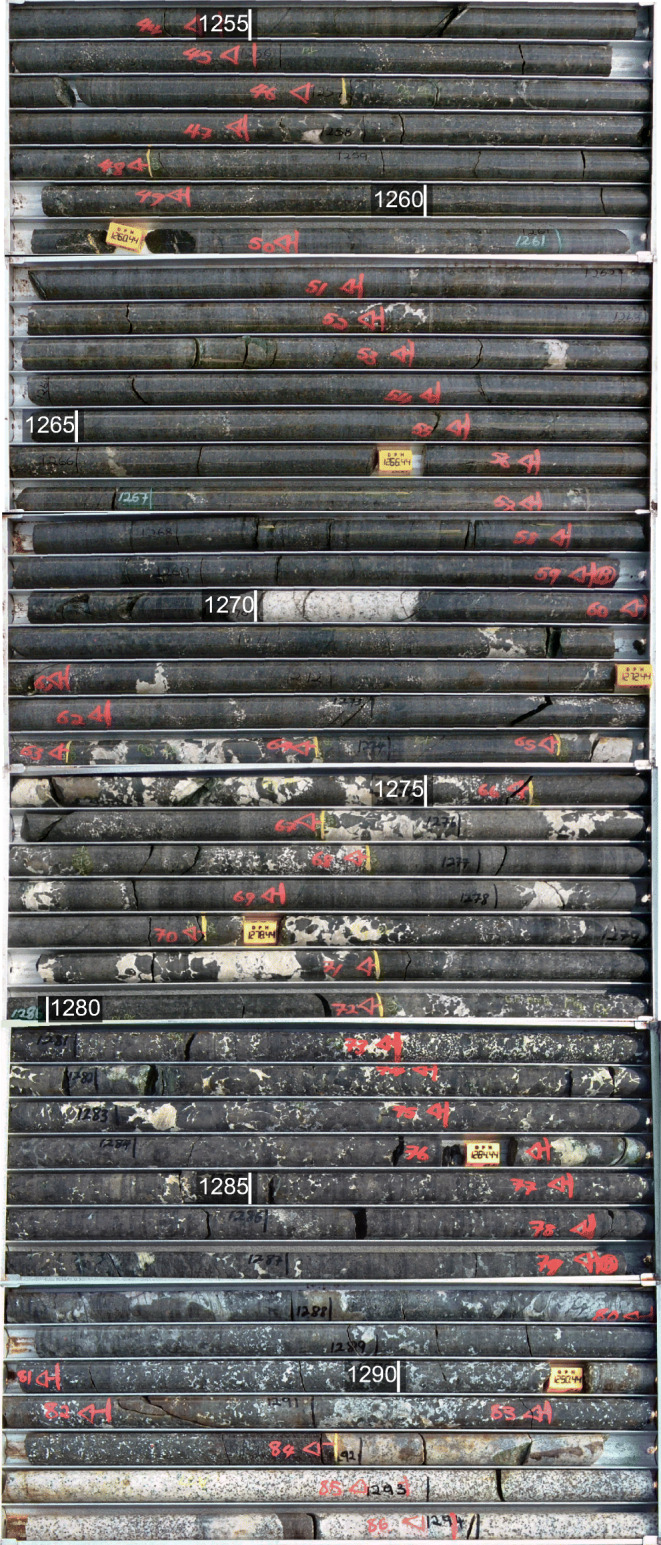
Pictures of core boxes from UMT094 showing lithologies and textural changes through the M2-M1_U_ sub-units and the contact with the FU at the base of the MU. Depth intervals every 5 m are indicated. The M2 sub-unit is a massive orthopyroxenite with small patches of pegmatoidal textures (e.g., near −1257 m and −1262 m). In contrast, pegmatoidal textures are dominant in the M1_U_ sub-unit (e.g., between −1274 and −1280 m) and alternate with non-pegmatoidal sections. The continuous white band at −1270 m is a granitic vein. The contact between the FU and MU at −1292 m is marked by a sharp contrast in color

Representative images of the most relevant lithologies analyzed are shown in Fig. 1. Pictures of the core boxes showing the transition between the M2 and M1_U_ sub-units and the contact between the MU and FU units are shown in Fig. 2. Gabbronorites of the Main Zone are the dominant lithologies to −1187 m below the surface, followed by mottled anorthosites (MAN), forming the hanging wall 2 (HW2) of the Bastard unit (BU). At −1214 m, interlayered norites and pyroxenites (HW1) appear but the contact with the MAN is gradational and some mottled anorthosites persist to −1216 m. Feldspathic pyroxenites appear at −1228 m and a thin (~ 1 cm) chromite stringer, considered part of the Bastard Reef (BAR) is present at −1233.22 m. In addition to the presence of the chromitite seam, the BAR was defined using available Pt-Pd-Au assay data, which were consistently above 1 ppm between −1230 and −1239 m. Below the BAR, and forming the uppermost sub-unit of the MU, there are feldspathic pyroxenites of the Middling Unit (Md1) to −1252 m, followed by a mineralized feldspathic pyroxenite (M2) to −1271 m, and a mineralized pegmatoidal pyroxenite (M1_U_) to −1292 m. A thin chromite stringer occurs at −1254 m, but the boundary between M2 and M1_U_ was defined by the textural change to pegmatoidal textures. The M2 - M1_U_ boundary is not sharp, with some prominent pegmatoidal patches occurring in the M2 (at −1258 and −1262.5 m) and intervals lacking pegmatoidal textures in M1_U_ (Fig. 2). Below the M1_U_, there is a very thin interval (10 cm) containing olivine and matching the characteristics of the M1_L_ but the interval is too thin to be represented in the stratigraphic column. The contact between the melanocratic MU and the leucocratic FU is sharp at −1292.95 m. The FU contains pyroxenite-norite rhythmic units and feldspathic pyroxenites (FW3) to −1336 m, followed by olivine gabbronorites to −1338.15 m. The UG-2 Unit underlies the FU and is composed of a feldspathic pyroxenite (UG-2 hanging wall or UG2HW) to −1364.77 m above the chromitite seam interpreted as the UG-2 correlative (−1364.77 to −1365.42 m) followed by pyroxenite to harzburgite in the UG-2 footwall (UG2FW) to −1404.03 m. Evidence of interaction with country rocks increases with depth, starting with the appearance of few small xenoliths at −1280 and −1335 m, followed by macroscopic evidence of intense country rock assimilation below −1350 m with calcsilicates and serpentinization (Keir-Sage et al. 2020).

**Fig. 3:**
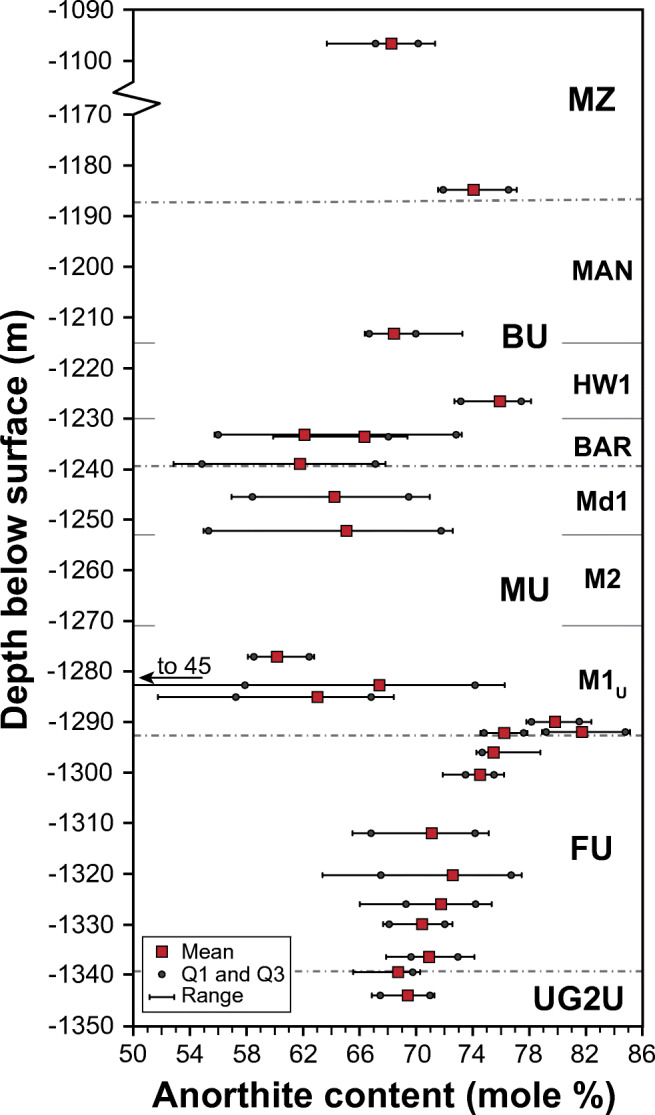
Variation in the anorthite content in plagioclase with depth in drill core UMT094. MZ = Main Zone, BU = Bastard Unit, MU = Merensky Unit, FU = Footwall Unit, UG2U = UG-2 Unit. Please refer to the text for the definition of sub-units

The major, minor, and trace element geochemical data (including Pt, Pd, and Au) for 50 samples collected between −1096 and −1402 m are reported in ESM1 Table 2. Assay data (Pt, Pd, Rh, Au, Ni, Cu, S, Cr) from −1215 to −1402 m are summarized in ESM1 Table 3. The plagioclase composition of 24 representative samples is documented in ESM1 Table 4, and the stratigraphic variations of the anorthite content in plagioclase are shown in Fig. 3. From the uppermost part of the UG2U to the top of the FU, there is a gradual increase in anorthite content from An_69_ to An_76_, with relatively small variations in An content within samples. Immediately above the FU, the highest An content (An_82_) was measured in plagioclase from the thin olivine-bearing sample at the base of the M1_U_ (sample UMT094-1291B), followed by plagioclase from the pegmatoidal pyroxenites immediately above (sample UMT094-1289) with An_80_. Within the rest of the MU and the BAR sub-unit of the BU, the median anorthite content fluctuates between An_60_ and An_69_. These intervals also yield the largest compositional ranges and uncertainties (e.g., from An_45_ to An_76_; An_(67 ± 12)_ in sample UMT094-1282). In the few analyzed samples located above the BAR, the anorthite content ranges between An_68_ and An_76_ showing relatively little intra-sample variation.

**Fig. 4:**
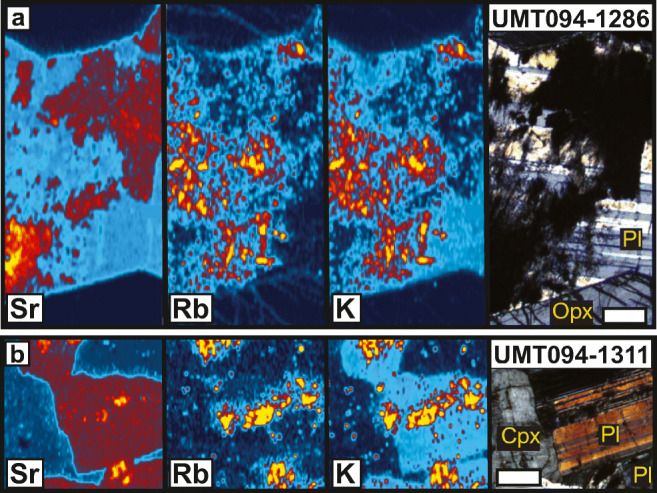
Element distribution maps showing examples of the effect of alteration on the content and distribution of Sr, Rb, and K in plagioclase. **a** Sample UMT094-1286. **b** Sample UMT094-1311. The maps show relative intensity within the mapped area in a warm-cold scale (i.e., yellow = highest relative content; dark blue = lowest). The cross-polarized images on the right are provided as reference and show the surrounding pyroxenes grains, the well-developed polysynthetic twinning in plagioclase and alteration patches. In the K maps, the light blue color indicates the K content of unaltered plagioclase and the yellow patches correspond to alteration. The white scale bars represent 0.5 mm

Because alteration patches were observed in some samples during petrographic analysis, the concentration of selected trace elements was determined in these samples. Figure 4 shows the K, Rb, and Sr content of plagioclase in one sample from the M1_U_ sub-unit of the MU (UMT094-1286) and one sample from the FU (UMT094-1311). The images in cross-polarized light show isotropic alteration patches within unaltered areas with clear polysynthetic twinning. Altered domains near the edges of plagioclase grains contain elevated Rb and K but no noticeable changes in Sr content. The dark alteration patches at the bottom left and top right of the plagioclase grain in Fig. 4a have the highest Sr content but lowest Rb and K content. Similar patterns can be seen in Fig. 4b. The Rb content is highest within the altered domain whereas unaltered plagioclase does not seem to contain more Rb than the surrounding clinopyroxene. The data indicate that even incipient alteration affects the Sr and Rb content, and therefore the Sr isotopic ratios of plagioclase. Therefore, careful petrographic observations prior to LA-MC-ICP-MS analysis were used to select only plagioclase grains with no evidence of alteration.

### In situ strontium isotopes in plagioclase

**Fig. 5:**
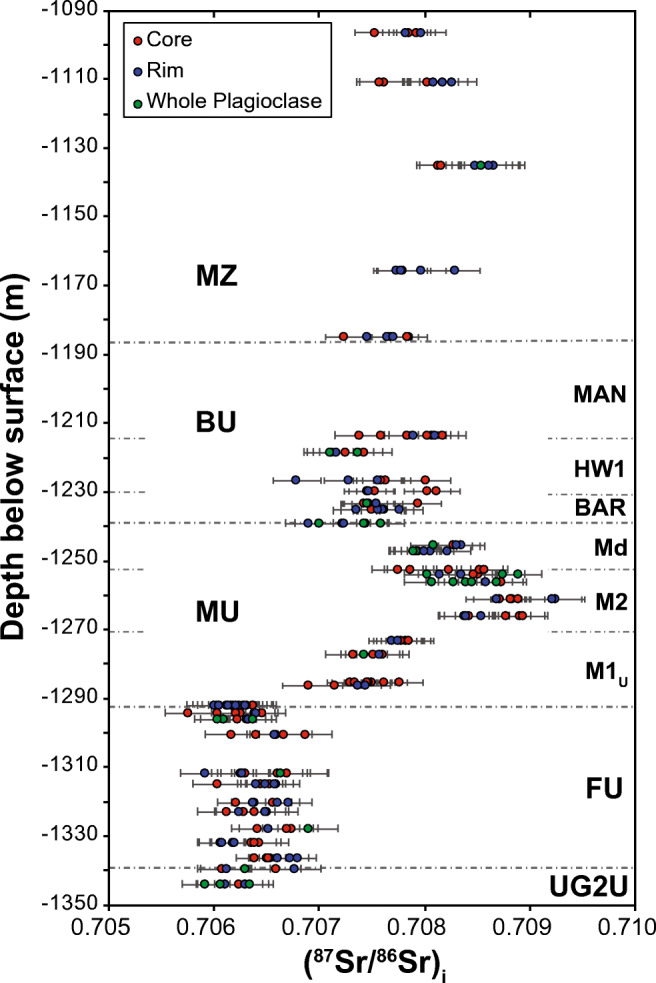
Stratigraphic variations in (^87^Sr/^86^Sr)_i_ throughout PGE-Ni-Cu mineralization in drill core UMT094. Uncertainties shown are the 1σ of each analysis

A total of 251 plagioclase analyses, including of core and rim domains of grains, were completed on 37 samples through the stratigraphy. The ^87^Sr/^86^Sr, ^87^Rb/^86^Sr, and (^87^Sr/^86^Sr)_i_ data are presented in ESM1 Table 5. The stratigraphic variations in Sr_i_ are shown in Fig. 5. Through the UG2U and FU, there is little variation (0.70575 < Sr_i_ < 0.70689; mean = 0.70636 ± 0.00024). However, within the M1_U_ sub-unit of the MU, and through roughly half the M2 sub-unit, Sr_i_ increases to up 0.70924 at −1260 m, then decreases gradually through the M2 and Md sub-unit. At the base of the BU (−1238 m) the Sr_i_ values range between 0.70690 and 0.70758 (mean = 0.70725 ± 0.00025). In contrast to the variations measured through the MU, there are no significant Sr_i_ shifts within the BU (0.70678 < Sr_i_ < 0.70816; mean = 0.70753 ± 0.00033). Values increase within the MZ from Sr_i_ = 0.70763 ± 0.00022 at the base of the MZ (−1184 m) to Sr_i_ = 0.70845 ± 0.00022 at −1135 m, then decrease to Sr_i_ = 0.70784 ± 0.00017 at −1096 m. In addition, isotopic data was collected on three altered plagioclase grains in sample UMT094-1286. The results (Sr_i_ = 0.71121 ± 0.00035) are consistent with the values obtained by Kruger 2005 (Sr_i_ = 0.7110 ± 0.0017) from plagioclase separates in samples from the shallow portion of the Platreef at Turfspruit.

## Discussion

### Strontium isotopic stratigraphy through mineralization in the northern limb of the BIC

**Fig. 6:**
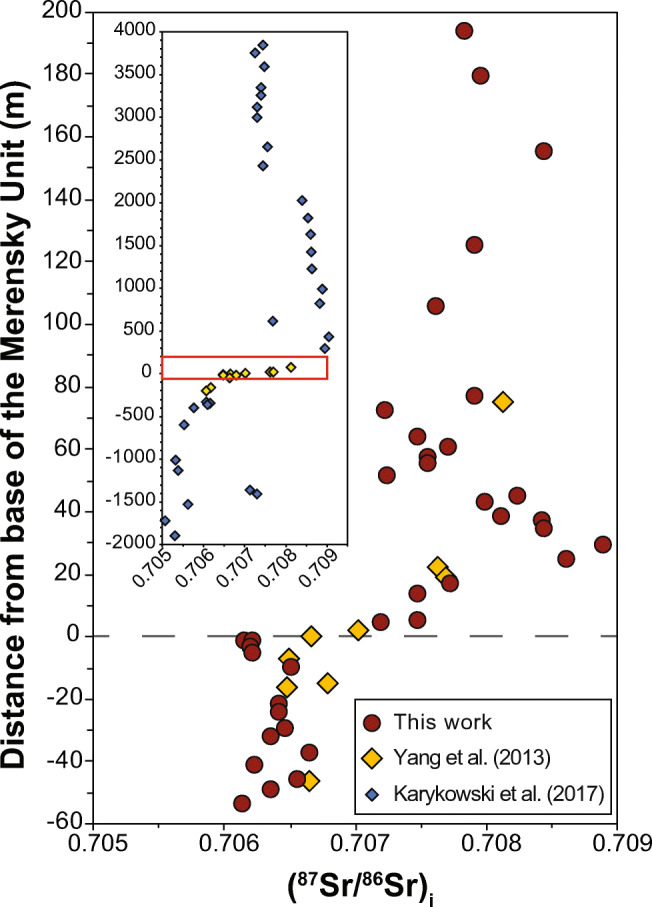
Comparison of the (^87^Sr/^86^Sr)_i_ stratigraphy thought the MU in the northern limb (this study) and the Union Section in the western limb (Yang et al. 2013). The inset highlights the relatively narrow stratigraphic interval where the major (^87^Sr/^86^Sr)_i_ shift occurs in the context of the Sr isotopic variations of the entire RLS (Karykowski et al. 2017). The profiles were matched using the start of Sr_i_ shift

**Fig. 7:**
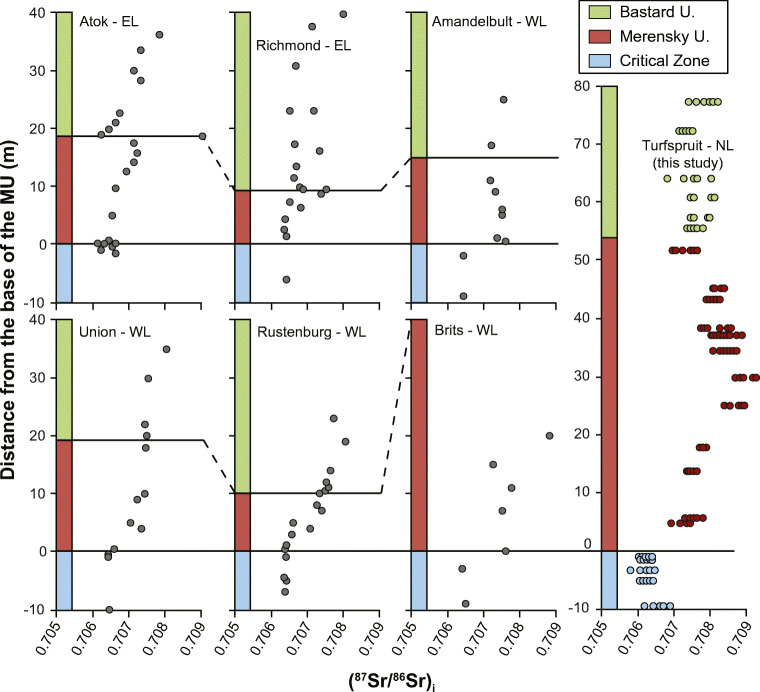
Comparison of (^87^Sr/^86^Sr)_i_ from six different sections of the eastern and western limbs (from whole rock analyses) with in situ (^87^Sr/^86^Sr)_i_ in plagioclase from the northern limb (this study). Eastern and western limb profiles after Seabrook et al. (2005)

To our knowledge, the Sr isotopic stratigraphic profile shown in Fig. 5 is one of the most detailed that have been documented through the Merensky Unit in the entire BIC. The (^87^Sr/^86^Sr)_i_ range from Sr_i_ = 0.70636 ± 0.00024 through the FU to a maximum of Sr_i_ = 0.70924, about 30 m above the base of the MU. This increase in Sr_i_ is consistent with the main characteristics of the MU first recognized in the western limb by Kruger and Marsh (1982). However, there are some significant differences. Figure 6 compares the Sr_i_ profile from the Flatreef (this work) with the Sr_i_ profile through the MU in the Union Section in the western limb (Yang et al. 2013), also obtained from in situ analyses on plagioclase. We are not aware of any other studies using in situ plagioclase analyses to document the Sr isotopic stratigraphy across the base of the MU in the eastern and western limbs. The base of the two profiles are almost identical, having essentially the same Sr_i_ values below the base of the MU and the same rate of change over the first 20 m above it. However, from a level about 20 m above, the base of the reef some significant differences can be inferred, despite the low data density in Yang et al. (2013). The inset in Fig. 6 shows the Sr_i_ stratigraphy through the Union Section (Karykowski et al. 2017), which expands on the work of Yang et al. (2013) and show that Sr_i_ values increase continuously to 300 m above the base of the MU. In contrast, the highest Sr_i_ values in our study were obtained from samples approximately 30 m above the base of the MU. From there, the Sr_i_ values decrease upwards to values around Sr_i_ = 0.708. The similarities in Sr_i_ below the inferred base of the MU and over the first 20 m of the MU validate the hypotheses of a correlation between PGE-Ni-Cu mineralization in the northern limb and the Merensky Unit in the eastern and western limbs of the BIC. Interestingly, a compilation of Sr isotope data from several profiles across the Merensky Reef in the eastern and western limbs (Seabrook et al. 2005) showed certain stratigraphic variations. Figure 7 shows the compiled Sr_i_ profiles in Seabrook et al. (2005) and the profile in this study. The Sr_i_ values below the base of the MU are consistently near 0.7065 but the stratigraphic position of the main Sr_i_ shift to higher values occurs at different levels. For example, at Union and Rustenburg, the Sr_i_ values increase gradually over the first 10 m, tapering to ca. 0.7075 at Union and increasing to slightly above 0.708 at Rustenburg. At Amandelbult and Brits, the Sr_i_ shifts immediately to ca. 0.7075 at the base of the MU, remaining roughly constant above that stratigraphic level, except for one outlier approaching 0.709 at Brits, 20 m above the base of the MU. The two profiles from the eastern limb show no changes at the base of the MU (for 5 m at Richmond and for 10 m at Atok) followed by a slight increase in Sr_i_ to 0.7075 towards the top of the MU, followed by a drop in Sr_i_ to values below 0.707 at the base of the BU. Interestingly, the highest Sr_i_ value at Atok (Sr_i_ = 0.7090) corresponds to a sulfide-bearing pyroxenite immediately above the chromitite stringer that defines the base of the BU (Lee and Butcher 1990). However, the reasons for the different stratigraphic position of the Sr isotopic shift and the variations through the MU and BU are not clear and further studies are required.

### Stratigraphic variations in An content of plagioclase and other geochemical parameters

**Fig. 8:**
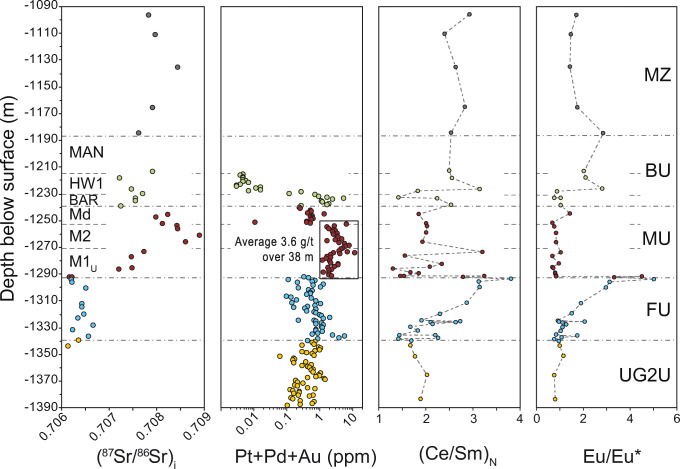
Comparison of stratigraphic variations in ^87^Sr/^86^Sr_i_, Pt + Pd + Au content, (Ce/Sm)_N_ and Eu/Eu*, obtained from whole rock data. Primitive mantle estimates for normalization are from Palme and O’Neill 2014. The box in the Pt-Pd-Au diagram highlights the high-grade interval, which contains an average of 3.6 ppm Pt + Pd + Au over 38 m (the combined M2 and M1_U_ thickness)

The increase in An content below the base of the MU (Fig. 3) is consistent with documented variations elsewhere in the RLS. For example, in the Union Section of the western limb (Karykowski et al. 2017; their Fig. 3) the An content in plagioclase is above An_70_ through most of the UCZ but drops below An_65_ near the transition to the MZ, at the same level at which there is a shift in Sr_i_. Another geochemical parameter of interest documented in Karykowski et al. (2017) is (Ce/Sm)_N_, which has constant values around 3 through most of the UCZ but is variable [1.5 < (Ce/Sm)_*N*_ < 4.5] at the stratigraphic level in which the Sr_i_ shift occurs. Figure 8 compares the stratigraphic variations in our study for Sr_i_, Pt + Pd + Au, (Ce/Sm)_N_, and Eu/Eu* (defined as 2xEu_N_/(Sm_N_ + Gd_N_). The Pt-Pd-Au values are significantly higher (average of 3.6 ppm over 38 m) over the stratigraphic interval in which the major shift in Sr_i_ occurs compared to the units above or below, which have Pt + Pd + Au contents consistently below 1 ppm. The whole rock (Ce/Sm)_N_ profile shows a trend of increasing values from roughly (Ce/Sm)_*N*_ = 2 through most of the FU to above (Ce/Sm)_*N*_ = 3 at the base of the MU, followed by a sharp decrease to roughly the same values documented for most of the FU. The Eu/Eu* profile shows a similar trend is more consistent through the MU and part of the BU than (Ce/Sm)_N_ values. Some of these variations could be attributed to the much lower plagioclase content throughout the MU and BU and the increasing proportion of plagioclase thought the intercalated pyroxenites-norites in the FU. However, the similarity with (Ce/Sm)_N_ variations near the top of UCZ in the Union Section (Karykowski et al. 2017) indicates that (Ce/Sm)_N_ could be a useful parameter to identify the MU and correlate lithologies between all the limbs of the RLS. The shift in Sr_i_ identified thought the Flatreef validates its correlation with the UCZ in the western and eastern limbs of the BIC, and the use of common terminology for the magmatic stratigraphy.

Identifying the source responsible for the Sr_i_ increase is more difficult. Karykowski et al. (2017) proposed that the isotopic shift documented in the Union Section of the western limb could be explained by mixing of UCZ and MZ magmas. This is a reasonable interpretation for the Union Section but not for the Flatreef because of the decreasing trend through the M2 and the lower Sr_i_ values through the BU and lower part of the MZ. A decrease in Sr_i_ at or near the base of the BU has been documented at the Atok and Richmond areas of the eastern limb, and to a lesser extent at the Amandelbult Section in the western limb (Fig. 7). However, one knowledge gap is the Sr_i_ of MZ rocks immediately above the MU and BU at those locations. Without such information it is difficult to assess how representative of the entire BIC are the Sr_i_ values for the MZ documented in Karykowski et al. (2017).

As shown in Fig. 8, the increase in Sr_i_ occurs at the same level as the increase in PGE contents. The similarities in Sr_i_ with the MU in the rest of the BIC are strong indicators that assimilation of local country rocks was not involved in the genesis of the PGE-Ni-Cu mineralization in UMT094. Additional evidence is documented by Keir-Sage et al. (2020) who showed that δ^34^S values through the MU, from samples in the same drill core, match the values documented for the eastern and western limbs. As shown in Grobler et al. (2019), there are no major geological structures between UMT094 the rest of the Flatreef and the surface exposure of the Platreef to the east. Also, as shown by van der Merwe (1976) and Ashwal et al. (2005) there are no major geological structures north of the Planknek Fault. Therefore, it is reasonable to assume that the magmatic sequences identified in the Flatreef are representative of the magmas in most of the northern limb (south of the Hout River Shear Zone), prior to interaction with local country rocks. This is also consistent with the presence of Merensky Reef and UG-2 correlatives south of the Planknek Fault (van der Merwe 2008; Maier et al. 2008). If the MU-BU sequence is bounded by unconformities with the rest of the UCZ below and the Main Zone above, and local contamination was not the trigger and source of mineralization, there is not enough magma volume in the emplaced sequence to extract PGE at the grades measured. Therefore, PGE enrichment most likely occurred at depth in a staging chamber, as in the “model 2” summarized in Maier et al. (2020).

### Future work

As shown in this study, the stratigraphic shifts in Sr_i_ are markers of the Merensky Unit and associated PGE-Ni-Cu mineralization not only in the eastern and western limbs of the BIC but also in the northern limb. However, the current framework to interpret these Sr isotopic shifts is insufficient. At present, the combined work of Yang et al. (2013) and Karykowski et al. (2017) are the best frame of reference for the Sr_i_ stratigraphy through mineralization in the Union Section of the western limb and this study is the best frame of reference for the Sr isotopic stratigraphy in the northern limb. The discrepancy in the Sr_i_ profiles through the UCZ-MZ transition is difficult to explain but scarce data available for other locations (Seabrook et al. 2005) indicate that there may be significant variability through the BIC. Thus, similar reference sections (from the immediate MU footwall through at least the first 100 m of the base of the MZ) are needed to constrain the variability in Sr_i_ across the entire BIC and test, for example, the hypothesis of mixing between UCZ and MZ magmas. Key localities include those for which there is incomplete data such as Atok, Richmond in the eastern limb as well as Rustenburg, Brits, and Amandelbult in the western limb. Key localities in the northern limb should include the Grasvally and Rooipoort areas where van der Merwe (2008) and Maier et al. (2008) identified Merensky and UG2 units.

## Conclusions

A detailed Sr isotope stratigraphy was established through a mineralized and uncontaminated section of the Flatreef and its immediate footwall and hanging walls in the Turfspruit farm near Mokopane. The results show a significant increase in (^87^Sr/^86^Sr)_i_ = Sr_i_ at the base of the main mineralized interval (from Sr_i_ = 0.7064 to a maximum of Sr_i_ = 0.7089). The results are consistent with the interpretation of these rocks as correlatives of the Merensky Unit and Bastard Unit in the eastern and western limbs of the BIC (Grobler et al. 2019). The increase in Sr_i_ values in UMT094 is preceded by an increase in the An content in plagioclase, from An_69_ to An_76_, and increases in whole rock Eu/Eu* and (Ce/Sm)_N_, similar to patterns that have been documented elsewhere in the BIC. These results are interpreted to indicate that the magmas that generated the PGE-Ni-Cu mineralization in the Flatreef, and by extension the Platreef, had the same sources as the magmas generating PGE-Ni-Cu mineralization in the Merensky reef of the eastern and western limbs. In addition, the Sr_i_ profile is interpreted as evidence that mineralization processes in the Flatreef are primordially magmatic and do not require interaction with immediate country rocks at the level of emplacement. Thus, the Flatreef was likely caused by the same processes that generated the Merensky Reef. Petrogenetically, the decrease in Sr_i_ through the M2 sub-unit to consistently lower Sr_i_ values through the BAR and lower part of the MZ indicate that the component causing the Sr_i_ increase through the Merensky Unit is not related to the Main Zone.

## Electronic supplementary material

ESM 1 (XLSX 98 kb)

ESM 2 (PDF 258 kb)
