# Supplementary material for: Strontium isotope stratigraphy through the Flatreef PGE-Ni-Cu mineralization at Turfspruit, northern limb of the Bushveld Igneous Complex: evidence of correlation with the Merensky Unit of the eastern and western limbs
Source: Miner Depos. 2020 Aug 20;56(1):59–72. doi: 10.1007/s00126-020-01006-3 (PMC7831340; doi:10.1007/s00126-020-01006-3)
Supplement: Supplementary file 2 — (PDF 258 kb) [file 126_2020_1006_MOESM2_ESM.pdf]

# Strontium isotope stratigraphy through the Flatreef PGE-Ni-Cu mineralization at Turfspruit, northern limb of the Bushveld Igneous Complex: Evidence of correlation with the Merensky Unit of the eastern and western limbs

Cédric C. Mayer · Pedro J. Jugo · Matthew I. Leybourne · Danie F. Grobler · Alexandre Voinot

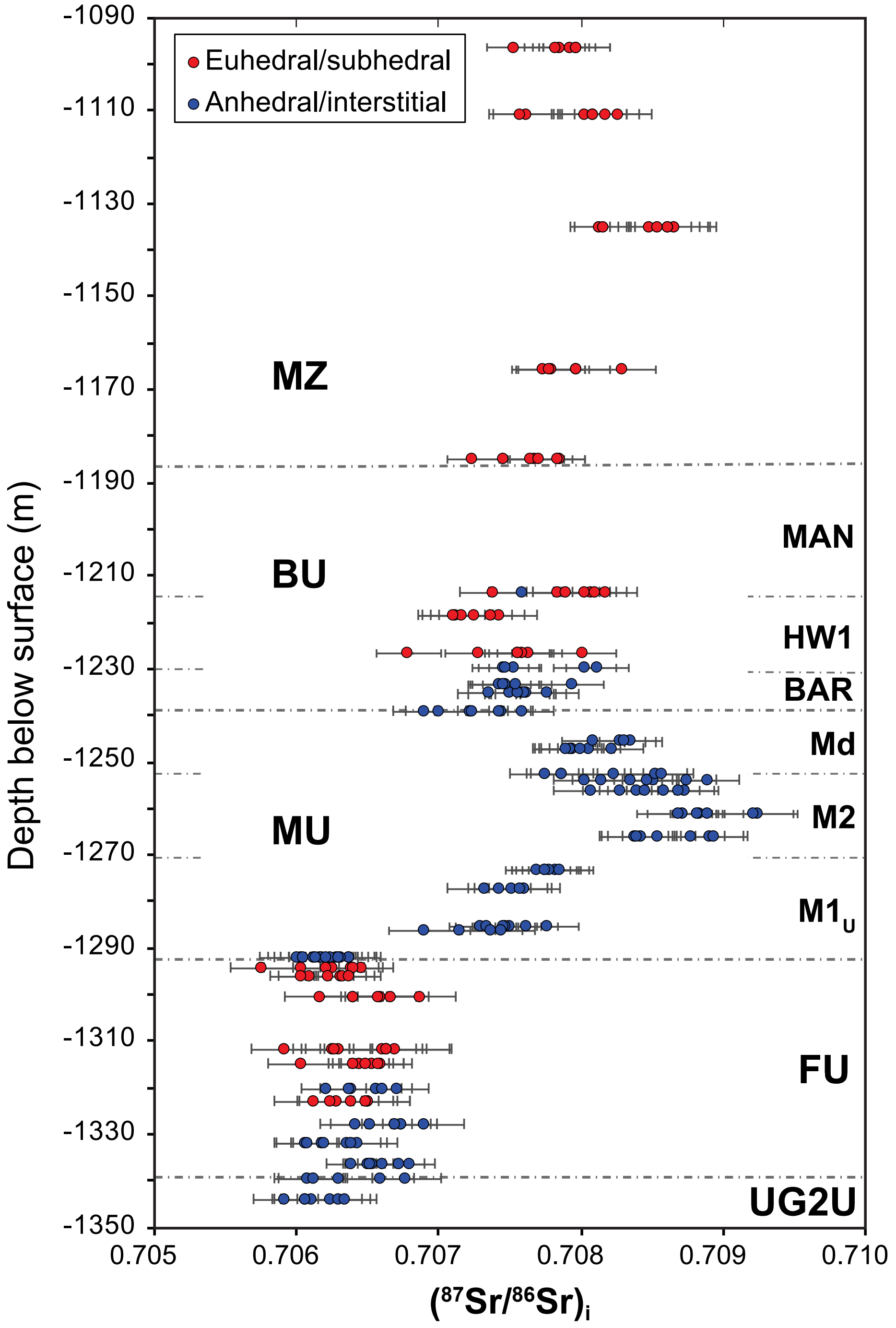


**Fig. ESM-1** Stratigraphic variations in ($^{87}Sr/^{86}Sr$)$_i$ coded by texture (euhedral/subhedral vs. interstitial). All analyzed plagioclase grains in the MU and in the BAR sub-unit of the BU are interstitial. Samples from the FU (with roughly equal amounts of samples for each textural type) show no differences in $Sr_i$ values.
